# Examining avian influenza virus exposure in seabirds of the northwest Atlantic in 2022 and 2023 via antibodies in eggs

**DOI:** 10.1093/conphys/coaf010

**Published:** 2025-02-24

**Authors:** Angela McLaughlin, Jolene Giacinti, Sailendra Nath Sarma, Michael G C Brown, Robert A Ronconi, Raphaël A Lavoie, Margaret L Eng, Bridget Enright, Andrew S Lang, Ishraq Rahman, Jordan Wight, Kathryn E Hargan, Mark L Mallory, Julia E Baak, Megan Jones, Michelle Saunders, Reyd Dupuis-Smith, Kyle Elliott, H Grant Gilchrist, Holly Hennin, Magella Guillemette, Pauline Martigny, William Montevecchi, Aevar Petersen, Yohannes Berhane, Jennifer F Provencher

**Affiliations:** Science and Technology Branch, Environment and Climate Change Canada, 1125 Colonel By Drive, Racen Road, Ottawa, ON, K1S 5B6, Canada; Bioinformatics, University of British Columbia, 315- 2185 East Mall, Vancouver, BC, V6T 1Z4, Canada; Science and Technology Branch, Environment and Climate Change Canada, 1125 Colonel By Drive, Racen Road, Ottawa, ON, K1S 5B6, Canada; Science and Technology Branch, Environment and Climate Change Canada, 1125 Colonel By Drive, Racen Road, Ottawa, ON, K1S 5B6, Canada; Canadian Wildlife Service, Environment and Climate Change Canada, 351 Saint-Joseph Boulevard, Gatineau, QC, K1A 0H3, Canada; Canadian Wildlife Service, Environment and Climate Change Canada, 45 Alderney Dr, Dartmouth, NS, B2Y 2N6, Canada; Science and Technology Branch, Environment and Climate Change Canada, 1550 d'Estimauville Ave, Québec, QC, G1J 0C3, Canada; Science and Technology Branch, Environment and Climate Change Canada, 45 Alderney Dr, Dartmouth, NS, B2Y 2N6, Canada; Science and Technology Branch, Environment and Climate Change Canada, 1125 Colonel By Drive, Racen Road, Ottawa, ON, K1S 5B6, Canada; Department of Biology, Memorial University of Newfoundland, 230 Elizabeth Ave, St. John’s, NL, A1B 3X9, Canada; Department of Biology, Memorial University of Newfoundland, 230 Elizabeth Ave, St. John’s, NL, A1B 3X9, Canada; Department of Biology, Memorial University of Newfoundland, 230 Elizabeth Ave, St. John’s, NL, A1B 3X9, Canada; Department of Biology, Memorial University of Newfoundland, 230 Elizabeth Ave, St. John’s, NL, A1B 3X9, Canada; Department of Biology, Acadia University, 15 University Ave, Wolfville, NS, B4P 2R6, Canada; Canadian Wildlife Service, Environment and Climate Change Canada, 933 Mivvik St, Iqaluit, NU, X0A 0H0, Canada; Department of Pathology and Microbiology, University of Prince Edward Island, 550 University Ave, Charlottetown, PEI, C1A 4P3, Canada; Department of Lands and Natural Resources, Nunatsiavut Government, 25 Ikajuktauvik Rd, Nain, NL, A0P 1L0, Canada; Department of Biology, Carleton University, 1125 Colonel By Drive, Ottawa, ON, K1S 5B6, Canada; Department of Natural Resource Sciences, McGill University, 21,111 Lakeshore Road, Montréal, QC, H9X 3V9, Canada; Science and Technology Branch, Environment and Climate Change Canada, 1125 Colonel By Drive, Racen Road, Ottawa, ON, K1S 5B6, Canada; Science and Technology Branch, Environment and Climate Change Canada, 1125 Colonel By Drive, Racen Road, Ottawa, ON, K1S 5B6, Canada; Département de Biologie, Université du Québec à Rimouski, 300 allée des Ursulines, Rimouski, QC, G5L 3A1, Canada; Département de Biologie, Université du Québec à Rimouski, 300 allée des Ursulines, Rimouski, QC, G5L 3A1, Canada; Department of Biology, Memorial University of Newfoundland, 230 Elizabeth Ave, St. John’s, NL, A1B 3X9, Canada; Brautarland 2, 108 Reykjavik, Iceland; National Centre for Foreign Animal Disease, Canadian Food Inspection Agency, 1015 Arlington Street, Winnipeg, MB, R3E 3M4, Canada; Science and Technology Branch, Environment and Climate Change Canada, 1125 Colonel By Drive, Racen Road, Ottawa, ON, K1S 5B6, Canada

**Keywords:** Antibodies, avian influenza virus, birds, H5n1, surveillance, wildlife monitoring

## Abstract

Seabirds are frequently infected by avian influenza virus (AIV), which prior to 2021 primarily consisted of low-pathogenic AIV with limited reports of disease during infection. However, since highly pathogenic AIV (HPAIV) H5N1 clade 2.3.4.4b was introduced to North America in late 2021, HPAIV outbreaks in seabirds have occurred in multiple regions, with high levels of morbidity and mortality in many species. While monitoring active viral infections is critical for tracking disease burden, monitoring prior viral exposure via antibody detection in species that experienced large outbreaks is important for identifying individual- and population-level impacts of AIV on immunity and survival. We capitalized on ongoing egg collection programmes to assess the prevalence of antibodies against AIV nucleoprotein (NP) and hemagglutinin subtype 5 (H5) in 523 eggs collected in 2022 and 2023 from 11 seabird species that breed in the northwestern Atlantic, including primarily samples from eastern Canada and two from western Iceland. The prevalence of AIV antibodies in eggs varied across regions, species and years. American common eider (*Somateria mollissima dresseri*) eggs had the highest AIV antibody prevalence compared to sympatric species in 2023. Longitudinal samples were available for northern gannets (*Morus bassanus*) and American herring gulls (*Larus argentatus smithsoniansus*) at several sites, where the prevalence of anti-NP and anti-H5 antibodies increased from 2022 to 2023. Examining AIV antibody prevalence in seabird eggs can be a useful tool to investigate population-level AIV exposure, while we acknowledge our limited understanding of differential antibody waning rates and the relationship between titre and susceptibility.

## Introduction

Seabirds are one of the reservoirs of avian influenza viruses (AIV), which prior to 2021 nearly entirely comprised low pathogenic AIV (LPAIV) with limited morbidity and mortality (Lang *et al.*, 2016). The H5N1 clade 2.3.4.4b was introduced to North America via the Atlantic and Pacific flyways following a significant European epizootic (Alkie *et al.*, 2022; Ramey *et al.*, 2022; Alkie *et al.*, 2023; Rahman *et al.*, 2024), resulting in elevated disease and mortality in wild birds, marine mammals, domestic birds, cattle and terrestrial mammals (WOAH, 2025). Unlike previous H5Nx outbreaks, HPAIV has persisted in wild bird populations, with elevated morbidity and mortality rates in seabird species globally (Lane *et al.*, 2023; Avery-Gomm *et al.*, 2024). Given the ongoing widespread, broad host diversity and high mortality of HPAIV in wild birds, there is a growing need to understand the spatiotemporal extent of AIV and HPAIV exposure and its consequences in wild birds. Seabirds are of particular interest as they can mediate transcontinental virus incursions (Caliendo *et al.*, 2022), have an affinity for agricultural landscapes (Garthe *et al.*, 2022), interact with people in urban environments (de Faria *et al.*, 2022) and are hunted for subsistence and feathers (Denlinger and Wohl, 2001; Montevecchi *et al.*, 2007; Bédard *et al.*, 2008; Natcher *et al.*, 2012).

Introductions of clade 2.3.4.4b from 2021 to 2023 to North America have drastically altered disease ecology in seabirds (Banyard *et al.*, 2022; Lane *et al.*, 2023). Since 2022, variation in viral detection (Giacinti *et al.*, 2024a) and disease impacts at individual and population levels (Lane *et al.*, 2023; Avery-Gomm *et al.*, 2024) among species and regions have been well documented. In 2021, great skuas (*Stercorarius skua*) in the UK displayed unusually elevated H5N1-attributable mortality at several colonies, but few other colonial seabirds showed clinical signs of infection (Banyard *et al.*, 2022). In 2022, H5N1 was detected in dozens of seabird species in the North Atlantic, with mass mortality events reported for northern gannets (*Morus bassanus*), common murres (*Uria aalge*), common eiders (*Somateria mollissima*; Avery-Gomm *et al.*, 2024) and Sandwich terns (*Thalasseus sandvicensis;*  Rijks *et al.*, 2022). Local, regional and national reports of H5N1 in seabirds have highlighted how seabird species may be differentially exposed and susceptible to HPAIV.

Exposure to influenza A virus (IAV) results in cell- and antibody-mediated adaptive immune responses and immune memory that can aid in response to subsequent infections (Doherty *et al.*, 2009). Serum can be used to detect antibodies in wild birds, e.g. against the conserved nucleoprotein (NP) and variable hemagglutinin (HA) surface protein; however, obtaining blood samples from migratory bird species, including seabirds, can be challenging. Eggs are easier to collect and contain maternal antibodies packaged during egg formation that provide passive immune protection (van Dijk *et al.*, 2014). While the transfer of maternal antibodies has been detected in several species, only a few studies have evaluated the relationship between maternal and offspring antibody titres (Lamb *et al.*, 2024). In free-living mallards (*Anas platyrhynchos*), AIV antibodies were detected in 48% of females and 43% of their eggs (van Dijk *et al.*, 2014), suggesting antibodies in eggs can be a proxy measure of exposure in laying females. Antibody persistence can vary by antigen, as well. For example, anti-H5 antibodies waned approximately twice as rapidly as anti-NP antibodies in a wild duck population in eastern Canada, which became H5 seronegative in 6 months while anti-NP antibodies were still detected (Wight *et al.*, 2024). Similarly, rapid waning of anti-H5 antibodies in relation to anti-NP antibodies was also observed in mallards (*A. platyrhynchos*) in southern Ontario, Canada (Giacinti *et al.*, 2024b). Thus, it is important to consider maternal, antigenic and temporal factors when interpreting prior exposure across and within species.

The vast majority of AIV detections in wild birds over the last few decades have been LPAIV subtypes (Spackman *et al.*, 2007), with sparse detections of H5N8 clade 2.3.4.4 and reassortant H5N2 in 2014 along the Pacific flyway (Lee *et al.*, 2015). However, following introductions of HPAIV H5N1 clade 2.3.4.4b to North America in late 2021 to 2023, H5N1 became the predominantly detected subtype across thousands of seabirds in eastern Canada (Giacinti *et al.*, 2024a). There was limited spread of Eurasian-origin H5N5 2.3.4.4b sublineages introduced to North America in 2023 in seabirds and detected in terrestrial mesocarnivores (Erdelyan *et al.*, 2024). Therefore, in this study we assumed detection of anti-H5 antibody in eggs indicated prior maternal HPAIV exposure, as there were negligible LPAIV H5 detections from 2021 to 2023.

As part of several programmes aimed at monitoring contaminants in the environment, Environment and Climate Change Canada (ECCC) in partnership with Indigenous governments, academic institutions and community groups have established egg collection programmes that date back to the 1970s. The original goal of these programmes was to track environmental contaminants as part of ECCC’s responsibility to report within regulatory frameworks such as the Stockholm Convention (Elliott *et al.*, 2005; Bianchini *et al.*, 2022). As a result, ECCC’s National Wildlife Research Centre (NWRC) in Ottawa, Ontario, Canada receives hundreds of eggs from across Canada annually. In response to the HPAIV outbreak in 2022 in wild birds, ECCC prioritized the need to assess HPAIV exposure and survival in species experiencing mortality events, to better inform population susceptibility to future infections (Nalepa *et al.*, 2024). In response, a subset of eggs collected under targeted contaminant programmes was sampled to detect antibodies indicative of maternal exposure to AIV (anti-NP antibodies) and potentially HPAIV (anti-H5 antibodies).

We report the prevalence of anti-NP and anti-H5 antibodies in 523 eggs from 11 seabird species collected across 32 sites in the northwestern Atlantic including eastern Canada and Iceland in 2022 and 2023. We hypothesized that exposure to H5 HPAIV increased from 2022 to 2023 where longitudinal samples were collected. Further, we tested if there was elevated antibody prevalence in eggs of species known to be important AIV reservoirs prior to the 2021 outbreak in Canada (e.g. murres, eiders, gulls) compared to species where viral detections were limited (e.g. gannets, kittiwakes, fulmars; Giacinti *et al.*, 2024a). We also evaluated whether there was a significant latitudinal gradient in AIV antibody prevalence in eggs, where we expected seabirds occupying higher latitudes to have lower exposure to H5 subtypes. This analysis serves as a descriptive ecological AIV monitoring baseline for future comparisons, which additionally demonstrates the utility of studying egg antibodies for wildlife disease surveillance. It further illustrates a generalizable approach to disease monitoring using seabirds as a sentinel group to inform conservation risks through a One Health lens (Mackenzie and Jeggo, 2019).

## Materials and Methods

### Ethical declarations

Eggs were collected with the necessary federal, provincial, territorial and Indigenous government permits and permissions in Newfoundland and Labrador (NL), Nunavut (NU), Nova Scotia (NS), New Brunswick (NB), Quebec (QC) and Iceland. This included permits from the Canadian Wildlife Service (scientific permits: northern, Atlantic and QC regions), territorial and provincial permits (research permits: NL, NU), protected area permits (e.g. Prince Leopold Island), Indigenous regional government permits (Nunatsiavut Government) and Icelandic authorities (Supplementary Table S1). Additionally, eggs were collected under academic or federal animal care permits, as dictated by the lead partner that carried out the work at the breeding colony given the shared nature of the large-scale egg collection programme in eastern and northern Canada (Supplementary Table S1).

### Egg collection

While annual egg sampling programmes took place across Canada, in 2022 and 2023 we targeted programmes that collected eggs in eastern Canada where the vast majority of HPAIV infections in seabirds had been reported (Giacinti *et al.*, 2024a). This region has many seabird colonies with high species diversity, and several partners were already in place to collect eggs from colonies that experienced HPAIV outbreaks during the study period. Additionally, we worked with partners in Iceland to collect seabird eggs as part of an ongoing collaborative research programme comparing seabird ecology between Canada and Iceland.

A single egg was sampled per nest and two eggs were never collected from the same clutch. Total eggs collected at each colony varied based on sampling planned for contaminants studies and availability of nests with eggs during the site visit. We targeted 10–15 eggs for each species at each site. Samples were collected for northern gannets in 2023 at Bonaventure Island, QC, on the basis of at least one parent having ocular anomalies associated with H5 (Lane *et al.*, 2023). All eggs were individually bagged and marked with an identifier and placed within a padded shipping container (Supplementary Fig. S1). The egg cases were kept cool, but not frozen, and shipped to the NWRC or the Atlantic Region Canadian Wildlife Health Cooperative (CWHC) at the University of Prince Edward Island (UPEI) for yolk collection from the eggs. While the yolks sampled at the NWRC stayed there for further analyses, the yolk samples collected at UPEI were then shipped frozen to Memorial University of Newfoundland (MUN) for antibody testing.

There were 523 seabird eggs collected between 28 June 2022 and 22 July 2023 and tested for presence of AIV anti-NP antibodies, and 405 (77.4%) were also screened for anti-H5 antibodies (Supplementary Fig. S2). Eggs were collected from 11 seabird species in the taxonomic orders Charadriiformes (herring gull, *Larus argentatus smithsoniansus;* black guillemot*, Cepphus grylle;* great black-backed gull, *Larus marinus;* glaucous gull, *Larus hyperboreus;* Arctic or common tern, *Sterna* spp*.;* black-legged kittiwake, *Rissa tridactyla;* razorbill, *Alca torda;* thick-billed murre, *Uria lomvia*)*,* Anseriformes (common eider [including subspecies *S. m. dresseri*, *S. m. borealis*, *S. m. sedentaria* based on collection sites]), Suliformes (northern gannet) and Procellariiformes (northern fulmar, *Fulmarus glacialis*)*.* Eggs were collected across 32 sites, most of which were seabird colonies (Supplementary Fig. S3, Supplementary Table S1). Prince Leopold Island, NU, had the most eggs sampled (*n* = 59) of any colony. Where egg development data were recorded, most Charadriiformes and Anseriformes eggs collected had no visible development, whereas the few Procellariiformes eggs were at later development stages, with feather follicles or first down appearance (Supplementary Fig. S5).

### Egg processing

In a biosafety cabinet at room temperature, eggs were cracked, opened, inspected and assigned a developmental age (Supplementary Fig. S5). Eggs with a yolk that could be collected were subsampled (i.e. eggs that were developed beyond a yolk sac being visible were excluded). The remaining portions of the eggs were homogenized, processed and archived as part of contaminants studies.

Egg yolks were processed using a chloroform extraction procedure, where 200 μl egg yolk was homogenized with 200 μl phosphate buffered saline (PBS) by gentle vortexing. At NWRC, PBS pH 7.4 (Corning, USA) was used and at MUN, PBS pH 7.2 (Gibco, USA, Product # 20012027) was used. An equal volume (400 μl) of chloroform (Spectrum Chemical Meg Corp, USA) was added and mixed by gentle vortexing. The mixtures were centrifuged at 6000 $x$ g for 15 min, and the aqueous solution was removed and stored at −20°C for analysis.

### Detection of anti-NP and anti-H5 antibodies at the NWRC

Most samples (89% of 523) were processed at the NWRC (Supplementary Table S5), where anti-NP and anti-H5 antibodies were detected with commercial enzyme-linked immunosorbent assay (ELISA) kits, AsurDx™ IAV NP Antibodies ELISA and AsurDx™ IAV H5 Antibodies ELISA, from Biostone Animal Health (Spackman and Killian, 2020; Hochman *et al.*, 2023). All egg antibody extracts were tested for anti-NP, and samples positive for anti-NP antibodies were tested for anti-H5 antibodies also at NWRC. Kits were used according to the manufacturer’s instructions. Optical density at 450 nm (OD_450_) was converted to percentage inhibition according to the manufacturer. With the Biostone kits, samples were considered positive for anti-NP antibodies if percentage inhibition (PI) ≥50% in both duplicates, and positive for anti-H5 antibodies if PI ≥45% in both duplicates. For quality control of each plate, the mean OD_450_ of the negative control had to be ≥0.5 and the PI of the positive control had to be ≥55%, as per the manufacturer. Intra-assay agreement between replicates was quantified with Pearson correlation coefficients (Supplementary Fig. S6). The log–linear relationship between anti-H5 antibody concentration and OD_450_ for the positive control in a serial dilution was used to suggest a tentative lower limit of detection and PI threshold (Supplementary Fig. S7).

### Detection of anti-NP antibodies at MUN

There were 57 samples screened for antibodies at MUN (Supplementary Table S5) where AIV anti-NP antibodies were detected using the IDEXX AI MultiS Screen Ab test (IDEXX Canada, Product # 99–12 119) as per manufacturer’s instructions (Brown, 2010) similarly to (Wight *et al.*, 2024). A sample to negative control (or signal to noise) ratio (S/N) <0.5 was considered positive for anti-NP antibodies.

### Detection of anti-NP and anti-H5 antibodies at NCFAD

All samples that tested positive for anti-NP antibodies at MUN were sent to the Canadian Food Inspection Agency’s (CFIA) National Centre for Foreign Animal Disease (NCFAD) to screen for presence of anti-H5 antibodies (Hochman *et al.*, 2023), as well as confirm detection of anti-NP antibodies for six samples from northern gannets on Bonaventure Island (Supplementary Table S5), which showed 100% agreement with the IDEXX kit. Anti-NP and anti-H5 antibodies at NCFAD were detected using previously described methods (Yang *et al.*, 2008), applying thresholds of average PI >30 for anti-NP antibodies and >35 for anti-H5 antibodies.

### ELISA comparison

We evaluated the agreement between ELISA protocols/kits with 26 egg samples screened using all of the Biostone, IDEXX and NCFAD anti-NP antibody protocols, and Biostone and NCFAD anti-H5 antibody protocols (Fig. 5). For both anti-NP and anti-H5 antibodies, we quantified the sensitivity and specificity of IDEXX and NCFAD assays relative to Biostone kits, which were chosen as the standard because they were most predominantly used in the study.

### Data curation

Metadata included species, taxonomic order, developmental age (Level 1–20), sample location (latitude/longitude, province/territory/country), sample collection date, processing date and ELISA results. We ensured sample collection dates were complete, IDs were unique and latitude and longitude for sampling sites were complete and accurate to three decimal points.

### Statistical analyses

Confidence intervals for percentage positive antibody detections (i.e. antibody prevalence) were calculated using one-group two-sided proportions tests (alpha = 0.05). Differences in antibody prevalence between groups were tested using Fisher’s exact tests, as most comparisons violated the chi-squared test assumption that >80% of expected frequencies exceed five (McHugh, 2013) and were underpowered for multivariate modelling. The relationships between latitude and prevalence of anti-NP antibodies and anti-H5 antibodies across colony sites were assessed using Pearson’s correlation coefficients and linear models for 2023, and individually for common eiders, black guillemots and herring gulls.

Analyses were developed using R version 4.3.2 (31 October 2023) and R packages tidyverse v1.3.2 (Wickham *et al.*, 2019), lubridate v1.8.0 (Grolemund and Wickham, 2011), cowplot v1.1.1 (Wilke, 2020), NatParksPalettes v0.2.0 (Blake, 2022) and pwr v1.3–0 (Champely, 2020).

### Mapping

Boundary files for Canada were obtained from the 2021 census as NAD83 Lambert datum (EPSG:3348; Statistics Canada, 2022), for the USA as WGS84 (USCB, 2019) and for Iceland and Greenland as WGS84 (gadm.org, 2022). The USA, Iceland and Greenland were reprojected as EPSG:3348. Sample latitude/longitude values in decimal degree were truncated to three decimal points, assigned the WGS84 (EPSG:4326) coordinate reference system, then reprojected to EPSG:3348. R packages used to generate maps included sf v1.0–8 (Pebesma, 2018), ggrepel v0.9.5 (Slowikowski, 2021), ggspatial v1.1.9 (Dunnington, 2023), magick v2.8.2 (Ooms, 2024) and plyr v1.8.7 (Wickham, 2011).

## Results

### Increased detection of AIV anti-NP and anti-H5 antibodies in eggs from 2022 to 2023

Across 523 samples, 383 eggs had detectable anti-NP antibodies (73.2%, 95% confidence interval one-group two-sided proportions test: 69.2–76.9%). All anti-NP-positive samples and 22 anti-NP-negative samples were also tested for anti-H5 antibodies, of which 25.7% were positive (21.6–30.3%, *n =* 104). In 2022, overall anti-NP antibody prevalence was 8.9% (2.9–22.1%, *n =* 45), but 0% (0–60.4%, *n =* 4) had detectable anti-H5 antibodies. By contrast in 2023, overall anti-NP antibody prevalence was 79.3% (75.3–82.8%, *n* = 478) and anti-H5 antibody prevalence was 25.9% (21.8–30.6%, *n* = 401). Samples in 2022 were limited to fewer species and locations.

**Figure 1:**
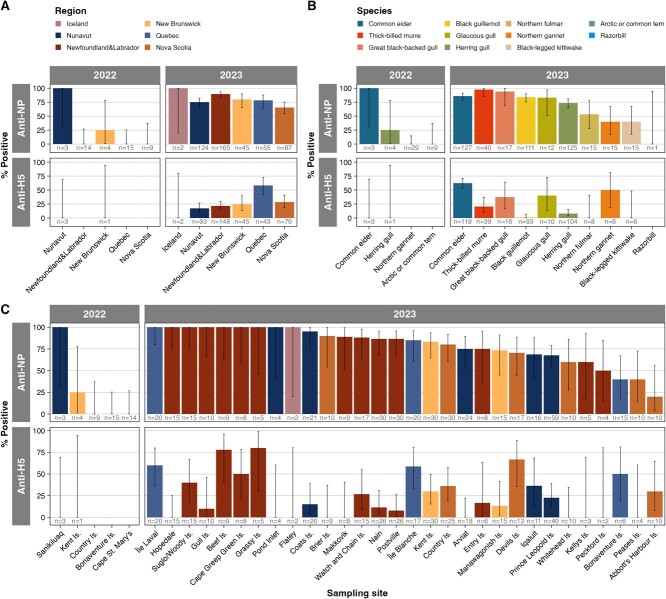
**AIV anti-NP and anti-H5 antibody prevalence in seabird eggs in 2022 and 2023**. Antibody prevalence (percent positive anti-NP and anti-H5) based on ELISA results by year, annotated with sample size across (**A**) regions (province/territory or Iceland), (**B**) species and (**C**) sites. Error bars represent 95% confidence intervals calculated using one-group two-sided proportions tests.

At the province/territory level (Fig. 1A; Supplementary Table S2), anti-NP antibody prevalence increased significantly from 2022 to 2023 in NL, NS, QC and NB (Fisher’s exact test; NL, NS, QC: *P* < 0.001, power = 99%; NB: *P* = 0.041, power = 46%). From 2022 to 2023, anti-H5 antibody prevalence increased in NB and NU, but the difference was not significant due to small sample size in 2022.

At the site level (Fig. 1C; Supplementary Table S3), anti-NP antibody prevalence increased from 2022 to 2023 on Country Island, NS (*P* < 0.001, power = 99%), Kent Island, NB (*P* = 0.033, power = 61%) and Bonaventure Island, QC (*P* = 0.02, power = 75%). In 2022, no anti-NP antibodies were detected on Country Island, NS, Bonaventure Island, QC and Cape St. Mary’s, NL, but otherwise anti-NP antibody prevalence ranged from 25% in Kent Island, NB (1.3–78.1%, *n* = 4) to 100% of samples in Sanikiluaq, NU (31–100%, *n* = 3). In 2023, anti-NP antibodies were detected at all sites except Cape St. Mary’s (all northern gannets) and eight sites were 100% anti-NP antibody positive (Fig. 1C; Supplementary Table S3). Overall, median anti-NP antibody prevalence across sites was 0% in 2022 (62.5% excluding zeroes) and 85.8% in 2023. No anti-H5 antibodies were detected in 2022 at any of five sites, including four samples that were anti-NP antibody positive from Kent Island, NB, and Sanikiluaq, NU. In 2023, seven sites had 0% anti-H5 antibody prevalence, and 23 sites had non-zero anti-H5 antibody prevalence from 7.7% in Postville, NL (1.3–26.6%, *n* = 26) to 80% on Grassy Island, NL (29.9–98.9%, *n* = 5). The median anti-H5 antibody prevalence across sites was 0% in 2022 and 15.8% in 2023 (33% excluding zeroes).

Herring gull, northern gannet and common eider eggs were collected in both 2022 and 2023 (Fig. 1B; Supplementary Table S4). In herring gulls, anti-NP and anti-H5 antibody prevalence increased from 2022 to 2023, but comparisons were not significant and underpowered (anti-NP antibodies, *P* = 0.066; anti-H5 antibodies, *P* = 1). Prevalence of anti-NP antibodies in herring gull eggs on Kent Island doubled (although underpowered to detect significance and data were generated using two different kits, Supplementary Table S5) from 25% (1.3–78.1%, *n* = 4) in 2022 to 50% (23.7–76.3%, *n* = 10) in 2023 (Supplementary Table S5). In gannets, anti-NP prevalence increased significantly from 2022 to 2023 (*P* < 0.001, power = 99%) and anti-H5 increased non-significantly. Common eider eggs had high anti-NP antibody detection in 2022 (100% of samples from Sanikiluaq, NU) and in 2023 (85.8, 78.3–91.2%, *n* = 127). Prevalence of anti-H5 antibodies in eiders increased non-significantly from 0% (0–69%, *n* = 3) in 2022 to 62.2% (52.8–70.8%, *n* = 127) in 2023 (*P* = 0.06, power = 35%).

### Species-level differences in AIV antibody prevalence in 2023

The prevalence of anti-NP antibodies in 2023 ranged from 40–97.5% across species, excluding one anti-NP-negative and anti-H5-negative razorbill (Fig. 1B; Supplementary Table S4). Eggs from thick-billed murres had the highest anti-NP antibody prevalence at 97.5% (85.3–99.9%, *n* = 40), followed by great black-backed gulls (94.1% (69.2–99.7%), *n* = 17), common eiders (85.8% (78.3–91.2%), *n* = 127), black guillemots (83.8% (75.3–89.9%), *n* = 111), glaucous gulls (83.3% (50.9–97.1%), *n* = 12), herring gulls (73.6% (64.8–80.9%), *n* = 125) and northern fulmars (53.3% (27.4–77.7%), *n* = 15), and antibody prevalence was <50% in northern gannets (40% (17.5–67.1%), *n* = 15) and black-legged kittiwakes (40% (17.5–67.1%), *n* = 15).

Common eider eggs had the highest anti-H5 antibody prevalence in 2023 (62.2% (52.8–70.8%), *n* = 119) of any species, followed by northern gannets (50% (18.8–81.2%), *n* = 6), glaucous gulls (40% (13.7–72.6%), *n* = 10), great black-backed gulls (37.5% (16.3–64.1%), *n* = 16), thick-billed murres (20.5% (9.9–36.9%), *n* = 39) and herring gulls (7.7% (3.6–15%), *n* = 104). The prevalence of anti-H5 antibodies was low in 2023 in black guillemots (1.1% (0.1–6.7%), *n* = 93), northern fulmars (0%, *n* = 8), black-legged kittiwakes (0%, *n* = 1) and razorbills (0%, *n* = 1).

**Figure 2:**
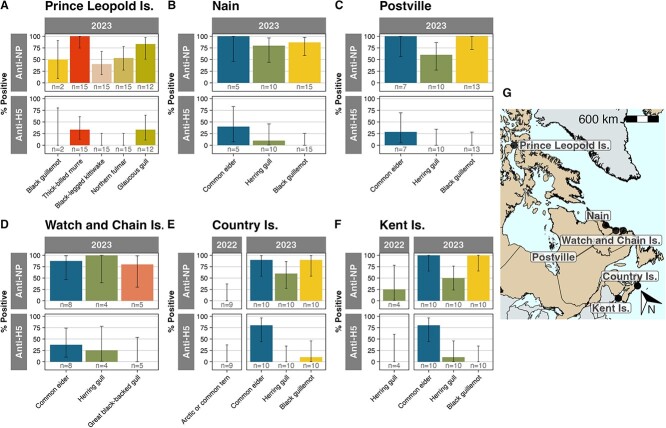
**AIV anti-NP and anti-H5 antibody prevalence in eggs by species and year for sites with three or more species sampled.** (**A**) Prince Leopold Island, Nunavut, (**B**) Nain, Newfoundland and Labrador, (**C**) Postville, Newfoundland and Labrador, (**D**) Watch and Chain Islands, Newfoundland and Labrador, (**E**) Country Island, Nova Scotia and (**F**) Kent Island, New Brunswick. (**G**) Sites with multiple species sampled. Error bars represent 95% confidence intervals calculated using one-group two-sided proportions tests.

Common eiders had among the highest anti-NP and anti-H5 antibody prevalence at multiple sites in 2023 (Fig. 2; Supplementary Table S3). Common eiders and black guillemots consistently had the highest anti-NP antibody prevalence compared to sympatric species on Country Island, NS, Kent Island, NB, Nain, NL, and Postville, NL (Fig. 2). At Watch and Chain Islands, NL, herring gulls had slightly higher anti-NP antibody prevalence (100% (39.6–100%), *n =* 4) than common eiders (87.5% (46.7–99.3%), *n* = 7), but sample sizes were small. Common eiders had the highest anti-H5 prevalence at Country Island, Kent Island, Nain, Postville and Watch and Chain Islands. At Prince Leopold Island, NU, thick-billed murres had 100% anti-NP antibody prevalence (74.7–100%, *n* = 15) and 33.3% (13–61.3%) anti-H5 antibody prevalence, while glaucous gulls had 83.3% (50.9–97.1%, *n* = 12) anti-NP antibodies and 33.3% (11.3–64.6%) anti-H5 antibodies, whereas black guillemots, black-legged kittiwakes and northern fulmars had moderate levels of anti-NP antibodies and no anti-H5 antibodies.

### Spatial differences in antibody prevalence

**Figure 3:**
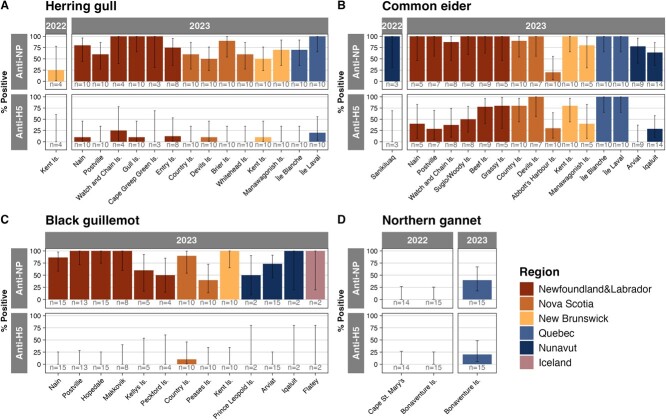
**AIV anti-NP and anti-H5 antibody prevalence in eggs across sites and year for species sampled from at least two sites.** (**A**) Herring gull, (**B**) common eider, (**C**) black guillemot and (**D**) northern gannet. Coloured by region (province/territory or Iceland). Error bars represent 95% confidence intervals calculated using one-group two-sided proportions tests.

Antibody prevalence varied across regions (Fig. 1A; Supplementary Table S2) and sites (Fig. 3; Supplementary Tables S3 and S5) in 2023. The highest regional anti-NP antibody prevalence was observed in Iceland (100%, 19.8–100%, *n* = 2), followed by NL (89.7% (83.8–93.7%), *n* = 165), NB (80% (64.9–89.9%), *n* = 45), QC (78.2% (64.6–87.8%), *n* = 55), NU (75% (66.3–82.1%), *n* = 124) and NS (65.5% (54.5–75.2%), *n* = 87). QC had the highest anti-H5 antibody prevalence (58.1% (42.2–72.6%), *n* = 43).

Site-level differences by species in 2023 were evaluated for herring gull, common eider, black guillemot and northern gannet eggs (Fig. 3A; Supplementary Table S5). For herring gulls in 2023, anti-NP antibody prevalence was 100% at 4 of 14 sites (Île Laval, QC, Watch and Chain Islands, NL, Gull Island, NL, and Cape Greep Green Island, NL), as well as 90% (54.1–99.5%, *n* = 10) at Brier Island, NS, and 80% (44.2–96.5%, *n* = 10) at Nain, NL. The highest anti-H5 antibody prevalence in herring gulls was detected in eggs from Watch and Chain Islands, NL (25% (1.3–78.1%), *n* = 4), followed by Île Laval, QC (20% (3.5–55.8%), *n* = 10). Seven sites had 0% anti-H5 antibody prevalence in herring gulls.

For common eiders in 2023, 9 of 15 sites had 100% anti-NP antibody prevalence (Fig. 3B; Supplementary Table S5). The lowest anti-NP antibody prevalence in common eiders was in Abbott’s Harbour Island, NS (20% (3.5–55.8%), *n* = 10) and Iqaluit, NU (64.3% (35.6–86%), *n* = 14). In contrast to herring gulls, common eider eggs had high anti-H5 antibody prevalence across sites, with 100% prevalence at three sites and four sites with 75–80% prevalence. Anti-H5 antibody prevalence was lower in common eider in NU with 0% (0–37.1%) in Arviat and 28.6% (9.6–58%) in Iqaluit.

Anti-NP antibodies in black guillemot eggs in 2023 were widely detected across sites (Fig. 3C) ranging from 40% (13.7–72.6%, *n* = 10) at Peases Island, NS, to 100% at all of Postville, NL (*n* = 13), Hopedale, NL (*n* = 15), Makkovik, NL (*n* = 8), Kent Island, NB (*n* = 10), Iqaluit, NU (*n* = 2), and Flatey, Iceland (*n* = 2). In contrast, anti-H5 antibodies were only detected in black guillemot eggs at Country Island, NS, where 10% (0.5–45.9%, *n* = 10) were anti-H5 antibody positive.

In 2022, northern gannet eggs had 0% anti-NP and anti-H5 antibody prevalence at Bonaventure Island, QC, and Cape St. Mary’s, NL (Fig. 3D). In 2023, anti-NP antibody prevalence in gannets at Bonaventure Island rose to 40% (17.5–67.1%, *n* = 15) and anti-H5 antibody positivity rose to 20% (5.3–48.6%, *n* = 15). All northern gannet data were generated using the IDEXX anti-NP antibody and NCFAD anti-H5 antibody ELISA protocols (Supplementary Table S5), with confirmatory NCFAD anti-NP antibody results for six samples in full agreement.

### AIV antibodies along latitudinal gradients

**Figure 4:**
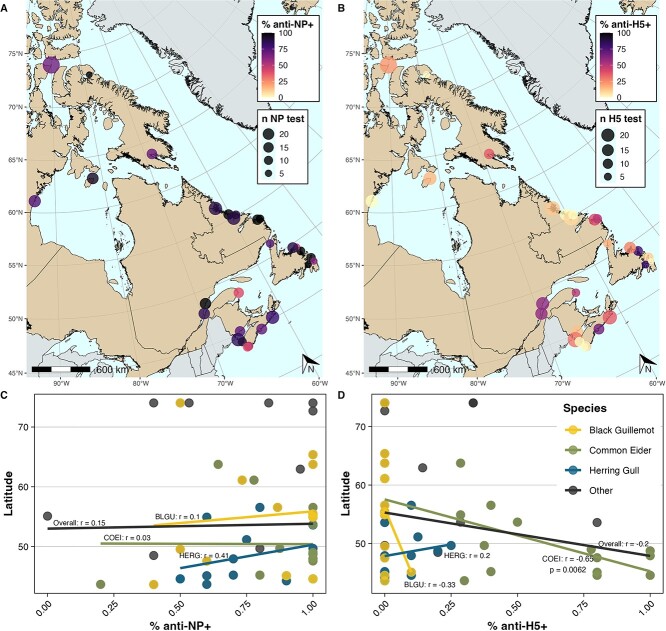
**Latitudinal trends in AIV antibody prevalence in seabird eggs in 2023 across sites**. Point size represents the number of samples tested and colour denotes overall prevalence of (**A**) anti-NP antibodies and (**B**) anti-H5 antibodies in 2023. Relationships between sites’ latitude and (**C**) anti-NP antibody or (**D**) anti-H5 antibody prevalence, overall and by species for common eider (COEI), herring gull (HERG) and black guillemot (BLGU). Pearson’s correlation coefficients (r) and, where significant, *P*-values were reported.

Latitude was not significantly correlated with anti-NP antibody prevalence across sites in 2023 overall or for key species (Fig. 4C). Pearson’s correlation coefficient (r) for the overall relationship between latitude and anti-NP antibody prevalence was 0.17 (*P* = 0.19). The correlation improved when only including herring gulls, but was not significant (r = 0.47; *P* = 0.07). There was a moderate inverse correlation of latitude and anti-H5 antibody prevalence overall (r = −0.17; *P* = 0.20; Fig. 4D), and when restricted to common eiders, there was a significant inverse correlation (r = −0.61, *P* = 0.011). In a linear model, anti-H5 antibody prevalence in common eiders was on average 35.5% (11.4–35.6%) lower for a 10-degree increase in latitude.

### Comparison of ELISA protocols

**Figure 5:**
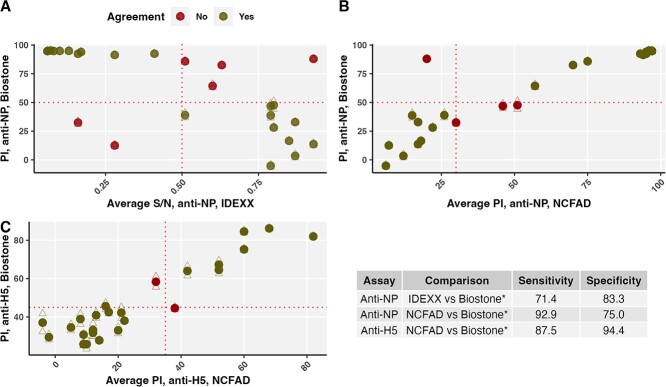
**Sensitivity and specificity of anti-NP antibody and anti-H5 antibody ELISA protocols.** Circles denote the mean PI and triangles are individual replicates for Biostone results on y-axes. Dashed lines indicate thresholds and separate quadrants of positive and negative detections. (**A**) The anti-NP antibody IDEXX kit (average sample/negative (S/N) ratio <0.5) compared to anti-NP antibody Biostone kit (PI ≥50 in both replicates). (**B**) Anti-NP antibody NCFAD (average PI >30) compared to Biostone. (**C**) Anti-H5 antibody NCFAD (average PI >35) compared to Biostone (PI ≥45 in both replicates). Sensitivity and specificity reported relative to Biostone kits.

For 26 egg samples, the sensitivity and specificity of the IDEXX and NCFAD anti-NP antibody and anti-H5 antibody NCFAD protocols were evaluated relative to the Biostone kits used for the majority of samples in the study (Fig. 5). For anti-NP antibodies, the sensitivity of the IDEXX kit relative to Biostone was 71.4% and the specificity was 83.3%, while the sensitivity of the anti-NP antibody NCFAD protocol was 92.9% and the specificity was 75.0%. For anti-H5 antibodies, the sensitivity and specificity of NCFAD relative to Biostone were 87.5 and 94.4%. Agreement between replicates in Biostone kits was high for these 26 samples (Supplementary Fig. S8) and in the main analysis (Supplementary Fig. S6).

## Discussion

Detection of AIV anti-NP and anti-H5 antibodies in eggs from 11 seabird species in the northwestern Atlantic varied by species, with common eider having the most elevated anti-H5 antibody prevalence across sites and years, suggesting variable maternal exposure. In 2022, anti-NP antibody prevalence was zero to low across multiple species (herring gulls, guillemots, terns, gannets) and sites, except for in eiders at Sanikiluaq, NU. Additionally, anti-H5 antibodies were not detected in any anti-NPantibody positive eggs, suggesting the maternal generation had no recent exposure to H5Nx HPAIV. The modest anti-NP antibody prevalence in eggs from herring gulls and common eiders in 2022 was unsurprising, as LPAIV circulates regularly in waterfowl and gulls (Olsen *et al.*, 2006).

No anti-NP antibodies were detected in eggs from northern gannets collected at Bonaventure Island, QC, and Cape St. Mary’s, NL, in 2022 (*n* = 29). Low immunity in gannets in 2022 is consistent with subsequently observed mass mortality events linked to HPAIV following the initiation of breeding by gannets in eastern Canada (Lane *et al.*, 2023; McPhail *et al.*, 2024). At Bonaventure Island, gannet eggs in 2023 had 40% anti-NP antibody prevalence and 20% anti-H5 antibody prevalence, corroborating that some individual gannets survived HPAI and laid eggs. Despite limited statistical power, these results suggest gannets in eastern Canada had limited exposure to AIV conferring a durable humoral immune response prior to the 2022 breeding season, and despite mass mortality events, some individuals mounted an antibody response to HPAIV, survived and went on to be active in the following breeding season. AIV antibody persistence in seabirds in the context of mortality and susceptibility should be considered in future studies.

Where species were sampled from the same colonies in both years, as for herring gulls at Kent Island, NB, and northern gannets at Bonaventure Island, QC, anti-NP and anti-H5 antibody prevalence increased from 2022 to 2023, suggesting HPAIV exposure increased during the study period and corroborating a widespread H5Nx outbreak, albeit sample sizes were low. The Kent Island herring gull colony experienced HPAI-related mortality in the middle of the 2022 breeding season (Taylor *et al.*, 2023) after the onset of egg laying, which would explain the absence of anti-H5 antibodies detected in 2022, and possible acquired immunity in 2023.

Eggs from thick-billed murres, black guillemots, herring gulls and great black-backed gulls had among the highest anti-NP antibody prevalence, but relatively low to moderate anti-H5 antibody prevalence, suggesting co-circulation H5 and non-H5 lineages prior to the breeding season in 2023, although this is confounded by potentially differential waning and assay sensitivity. Difference between anti-NP and anti-H5 antibody concentrations could be explained by co-circulating HA types, but could also be affected by more rapid waning of anti-H5 antibodies than anti-NP antibodies (Wight *et al.*, 2024; Giacinti *et al.*, 2024b).

Common eider eggs had high anti-NP and anti-H5 prevalence in 2023, suggesting widespread recent infection of eiders by H5Nx. Fulmar and kittiwake eggs from Prince Leopold Island, NU, in 2023 had moderate anti-NP antibody prevalence but zero anti-H5, suggesting recent LPAIV exposure. Differing exposure supports that while seabirds may share life history traits, breeding colonies, migration routes and foraging zones, their AIV exposure and immunity durability can differ (Wille *et al.*, 2014; Lang *et al.*, 2016; McPhail *et al.*, 2024).

We collected samples from several colonies where different species nest in close proximity (e.g. Country Island, NS, and Kent Island, NB), and in some cases directly overhead of each other (e.g. Prince Leopold Island, NU). Common eider eggs had the highest anti-NP and anti-H5 antibody prevalence at sites where multiple species were tested. At Prince Leopold Island in 2023, while thick-billed murre eggs had the highest prevalence of anti-NP and anti-H5 antibodies compared to other species, we also detected anti-NP and anti-H5 antibodies in glaucous gull eggs, but only anti-NP antibodies in the black guillemot, black-legged kittiwake and northern fulmar eggs, suggesting differential H5 exposure or antibody waning by species. At Country Island, NS, in 2023, common eiders and black guillemots had the highest anti-NP antibody prevalence followed by herring gulls, but while 80% of the common eider eggs were anti-H5 antibody positive, only 10% of guillemot eggs were anti-H5 antibody positive, and no herring gull eggs were anti-H5 antibody positive. At Kent Island, NB, a different pattern was observed among the same species. While the eiders again showed the highest anti-NP and anti-H5 antibody prevalence, the guillemot eggs had 100% anti-NP antibody prevalence, but 0% anti-H5 antibody prevalence, while herring gulls had lower anti-NP antibody prevalence (50%) but the second highest anti-H5 antibody prevalence (10%). Although limited in some instances by small sample size, these results highlight how sympatric nesting seabird species may be differentially exposed to AIV, confounded by potentially differential antibody waning rates, which is critical context for estimating the potential impacts on individuals and populations.

Colonies sampled in this study were spread over almost 30 degrees of latitude and we expected differences in antibody prevalence from north to south given that AIV detections have generally been less frequent in northern compared to southern Canada (McLaughlin *et al.*, 2024; Giacinti *et al.*, 2024a). We sampled common eiders from Abbott’s Harbour at the southern tip of NS, throughout the St. Lawrence and Atlantic region and as far north as Frobisher Bay, NU. This represents 16 colonies across several ecozones (coastal temperate to Arctic tundra), and across the range of the three subspecies of common eiders in eastern Canada (*S. m. dresseri* in Atlantic Canada including southern Labrador, *S. m. borealis* on Baffin Island and northern Labrador, and *S. m. sedentaria* in southern Hudson Bay; Goudie *et al.*, 2020). Prevalence of anti-NP antibodies was consistently ≥90% at most colonies, with anti-H5 antibody prevalence generally lower but up to 100% at three colonies (Île Blanche, QC, Île Laval, QC, and Devils Island, NS). The only site where anti-H5 antibodies were not detected in 2023 was Arviat, NU, on the west coast of Hudson Bay. There was no correlation between anti-NP antibody prevalence in common eiders and latitude, but a significant inverse correlation between anti-H5 antibody prevalence in common eiders and latitude. This suggests that while AIV exposure was common throughout the range of common eiders in Canada in 2023, anti-H5 antibodies were elevated in eiders in the south in 2023, possibly due to differences in migrations and winter distributions among subspecies, e.g. mixing among *dresseri* subspecies (Goudie *et al.*, 2020; Mallory *et al.*, 2020). Future work on AIV antibody prevalence in eggs should prioritize common eider egg samples from the same locations to examine these potential spatial trends over time.

We categorized prior exposure as LPAIV or HPAIV based on the presence of H5 antibodies for this study. We acknowledge that LPAIV H5 subtypes circulate naturally in wild birds (mostly ducks) and that not all HPAIV are H5 (for example, H7N7). However, since the 2021 H5N1 clade 2.3.4.4.b incursions to North America, up to 2024, the large majority of H5 detected has been H5N1 and to a lesser extent H5N5; LPAIV H5 was not detected over this period (Giacinti *et al.*, 2024a).

While agreement between ELISA protocols results was imperfect, sensitivity and specificity were moderately high for anti-NP antibodies (IDEXX and NCFAD compared to Biostone) and moderate for anti-H5 antibodies (NCFAD compared to Biostone). In the main body of this study, the IDEXX assay was used for a minority of samples, mostly those sampled in 2022. The only comparison for which this might have made a difference is for herring gulls sampled on Kent Island analysed using IDEXX/NCFAD in 2022 and Biostone in 2023, and where the anti-NP antibody prevalence appeared to double from 2022 to 2023, and anti-H5 antibodies went from 0 to 10%. If IDEXX anti-NP antibodies or NCFAD anti-H5 antibodies were more likely to identify false negatives, that could have partially accounted for the increases in antibody prevalence from 2022 to 2023. Most focal species of the analyses, such as common eider, were entirely evaluated using the Biostone kits. Only 57 eggs of 523 were analysed with the IDEXX anti-NP antibody kit, of which six samples had confirmatory NCFAD anti-NP antibody results reported. Alternative PI thresholds could lead to improved agreement between kits’ results. The threshold recommended for the anti-H5 antibody Biostone kit was close, albeit slightly more conservative compared to what we identified as the lower limit of detection based on linearity between OD and antibody standard concentration. Therefore, false negatives were possible in all assays. Further work on interpreting ELISA results as titres would improve our understanding of AIV exposure and susceptibility.

Sparse data from 2022 limits our ability to detect increased antibody prevalence from 2022 to 2023, and there were only three sites with data from both years. In the future, repeated sampling at the same sites will improve comparisons. In part this is due to a lack of samples, as the outbreak started 2022. Statistical power tests can inform the number of samples needed, which helps to mitigate sampling bias. Monitoring of active LPAIV and HPAIV infections via PCR detections in murres corroborates that there was a significant H5Nx HPAIV outbreak in the Atlantic region in June to August 2022 (McLaughlin *et al.*, 2024).

Non-random sampling limits the reliability of comparisons within this study. For instance, in 2022, northern gannet egg collection at Bonaventure Island was relatively random, but in 2023, sampling was semi-random, with a subset of eggs (6/15) targeted from nests where at least one parent had an ocular anomaly (change in coloration of one or both irises) that could indicate prior infection with H5N1. This type of lesion has been reported in wild and domestic ducks (Brown *et al.*, 2014; Yamamoto *et al.*, 2016,), and the probability of detecting H5-specific antibodies is higher in gannets with ocular anomalies (Lane *et al.*, 2023). All six gannet eggs from parents with an ocular anomaly were anti-NP antibody positive, but only 50% were anti-H5 antibody positive.

Other biases associated with egg collections must be considered, including convenience sampling since easily accessed eggs may be more likely to be sampled. Adults with poorer condition may nest in more exposed areas, making their eggs easier to access. This may apply to some species within this study that nest in dense colonies where only the edges are accessed by visiting researchers (e.g. murres, terns, gannets), but for most species represented here, all nest locations are accessible to egg collectors. Spatially clustered sampling bias, whereby eggs from proximate nests are more likely to be sampled, is also important to consider, and we tried to account for this by testing multiple sites/colonies. Survival bias should also be considered in the interpretation of antibody prevalence, as birds infected with highly virulent AIV resulting in death would not lay eggs or be detected in this study.

Egg laying order and female physical health during laying can affect maternally transferred antibodies. In captive mallards, yolk antibody concentration was higher in eggs laid later in the clutch (van Dijk *et al.*, 2014). However, in the long-lived colonial yellow-legged gull (*Larus michaehellis*), yolk anti-AIV antibody concentrations were lower in eggs laid later, and this was more pronounced in clutches laid by physically asymmetric females (Hammouda *et al.*, 2012). Also, asymmetric females in worse physical condition produced fewer antibodies and transferred lower amounts of antibodies to eggs (Hammouda *et al.*, 2012). During egg formation, serum IgY is selectively transferred to the yolk via a surface receptor on the yolk membrane, which may differ by species, whether hatching in a species is synchronous and the energy demands on the female during laying (van Dijk *et al.*, 2014). In toxicology, egg lay order influences contaminant concentrations in eggs, and by taking one egg per nest, at the scale of the study, egg order is not likely to be highly important as the sample represents a variety of eggs (Akearok *et al.*, 2010). For many seabirds, such as murres, fulmars, gannets and guillemots, only one or two eggs are laid, and hence this is not a concern. Egg order is likely only to be a confounding factor for species with larger clutches (e.g. eiders, gulls) or if the titre differential between eggs spanned the threshold to designate a sample as positive. Studies comparing antibody concentration in eggs and matched maternal plasma and Nobuto strips are needed and underway.

Our results need to be contextualized within the limitations of antibody assays. Two samples were anti-NP antibody negative and anti-H5 antibody positive (405 eggs tested for both), suggesting limitations in assay sensitivity, particularly as anti-H5 antibodies waned approximately twice as rapidly as anti-NP antibodies in a study of ducks (Wight *et al.*, 2024; Giacinti *et al.*, 2024b). This also highlights that testing samples for anti-H5 antibodies should not necessarily be predicated on anti-NP antibody positivity, especially for PI close to the threshold. Understanding production and waning of AIV antibodies in seabirds, in relation to time since outbreak and egg age, is a critical component to estimating their long-term immune response to currently circulating and future AIV subtypes (Wight *et al.*, 2024). Similarly, assessing the sensitivity and specificity for antibody assays of egg yolks is fundamental to broadly apply them for ecological questions and wildlife health surveillance. Captive studies of species that may be repeatedly sampled would help the interpretation of results in wild species where sampling is limited, such as seabirds that only come to land during their breeding season. However, having a consistent and narrow annual window of opportunity is a strength of conducting wildlife disease surveillance by detecting antibodies in seabird eggs. There were likely undetected false negatives due to waning or conservative PI thresholds. An additional caveat is that we detected antibodies in egg yolks using commercial ELISA kits developed to detect antibodies in plasma. Substrate effects or cross-reactivity with off-target egg antigens may be possible. Further optimization of antibody extraction methods from yolks could also be beneficial.

Almost all the eggs were from eastern and northern Canada, but the study also included two black guillemot eggs sampled from Iceland. More guillemot eggs were sampled in Iceland, but upon dissection, only two contained sufficient yolk. Iceland eggs were collected to provide an outgroup to eggs collected in eastern and northern Canada. While the sample size was small, we found similar levels of both anti-NP and anti-H5 antibodies in the guillemot eggs from Iceland compared to Arctic and Atlantic Canada, suggesting similar levels of pathogen exposure during the pre-breeding season. Regional comparisons are critical as many studies have shown that seabirds in the North Atlantic have a high degree of mixing during the non-breeding season, which has been hypothesized as a potential route for the transmission of AIV between continents (Caliendo *et al.*, 2022). Further collaborations should be undertaken by the seabird community to compare and contrast AIV immunity across regional scales in order to inform population-level risk assessments.

Key questions remain unanswered in relation to monitoring landscape-level AIV exposure through antibodies in eggs. It is yet unclear how AIV anti-NP and anti-H5 antibody concentrations relate to susceptibility to and disease burden from different AIV subtypes, as well as how original antigenic sin may impact AIV susceptibility in seabirds (Zhang *et al.*, 2019). Further research into susceptibility and antibody waning is needed to enhance our understanding of these dynamics.

We demonstrate that exposure to AIV generally and the most recent H5 HPAIV can be detected via AIV antibodies in wild migratory bird eggs, which represents a wide-scale, minimally invasive monitoring tool in wild birds. Pathogen surveillance in known AIV reservoir species will continue to be important to detect future outbreaks and assess risk. Additionally, regular monitoring of AIV exposure in other susceptible species can help inform discussions and mobilization of reactive monitoring. As eggs are collected annually in many regions for contaminant monitoring, detecting AIV and other pathogen antibodies in eggs is a low-cost, minimally invasive and effective tool for wildlife health programmes.

## Supplementary Material

Web_Material_coaf010

## Data Availability

The data underlying this article will be shared on reasonable request to the corresponding author.
