# Supplementary material for: Examining avian influenza virus exposure in seabirds of the northwest Atlantic in 2022 and 2023 via antibodies in eggs
Source: Conserv Physiol. 2025 Feb 24;13(1):coaf010. doi: 10.1093/conphys/coaf010 (PMC11858004; doi:10.1093/conphys/coaf010)
Supplement: Web_Material_coaf010 [file web_material_coaf010.pdf]

Supplementary Material

Examining avian influenza virus exposure in seabirds of the northwest Atlantic in 2022 and 2023 via antibodies in eggs

Angela McLaughlin[1,2], Jolene Giacinti[1], Sailendra Nath Sarma[1], Michael G.C. Brown[3], Robert A. Ronconi[4], Raphael A. Lavoie[5], Margaret L. Eng[6], Bridget Enright[1], Andrew S. Lang[7], Ishraq Rahman[7], Jordan Wight[7], Kathryn E. Hargan[7], Mark L. Mallory[8], Julia E. Baak[9], Megan Jones[10], Michelle Saunders[11], Reyd Dupuis-Smith[12], Kyle Elliott[13], H. Grant Gilchrist[1], Holly Hennin[1], Magella Guillemette[14], Pauline Martigny[14], William Montevecchi[7], Ævar Petersen[15], Jennifer F. Provencher[1]*

Affiliations
[1]Science and Technology Branch, Environment and Climate Change Canada, Ottawa, ON, Canada
[2]Bioinformatics, University of British Columbia, Vancouver, BC, Canada
[3]Canadian Wildlife Service, Environment and Climate Change Canada, Gatineau, QC, Canada
[4]Canadian Wildlife Service, Environment and Climate Change Canada, Dartmouth, NS, Canada
[5]Science and Technology Branch, Environment and Climate Change Canada, Québec, QC, Canada
[6]Science and Technology Branch, Environment and Climate Change Canada Dartmouth, NS, Canada
[7]Department of Biology, Memorial University of Newfoundland, St. John's, NL, Canada
[8]Department of Biology, Acadia University, Wolfville, NS, Canada
[9]Canadian Wildlife Service, Environment and Climate Change Canada, Iqaluit, NU
[10]Department of Pathology and Microbiology, University of Prince Edward Island, Charlottetown, PEI, Canada
[11]Department of Lands and Natural Resources, Nunatsiavut Government, Nain, NL, Canada
[12]Department of Biology, Carleton University, Ottawa, ON, Canada
[13]Department of Natural Resource Sciences, McGill University, Montréal, QC, Canada
[14]Département de Biologie, Université du Québec à Rimouski, Rimouski, QC, Canada
[15]Brautarland 2, Reykjavik, Iceland
[16]National Centre for Foreign Animal Disease, Canadian Food Inspection Agency, Winnipeg, MB, Canada

*Corresponding author – jennifer.provencher@ec.gc.ca

Supplementary Figures

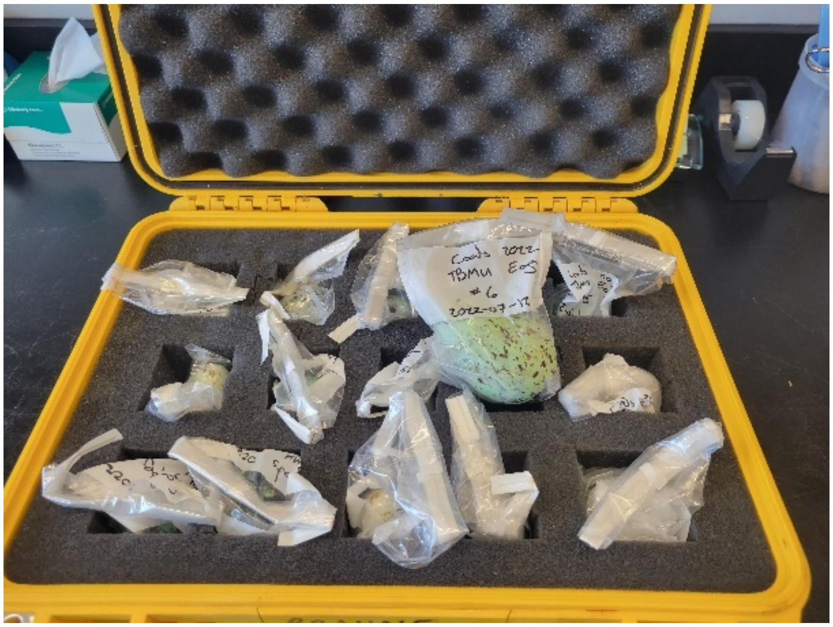

Fig. S1. Representative egg storage case for sample collection and delivery to processing centres.

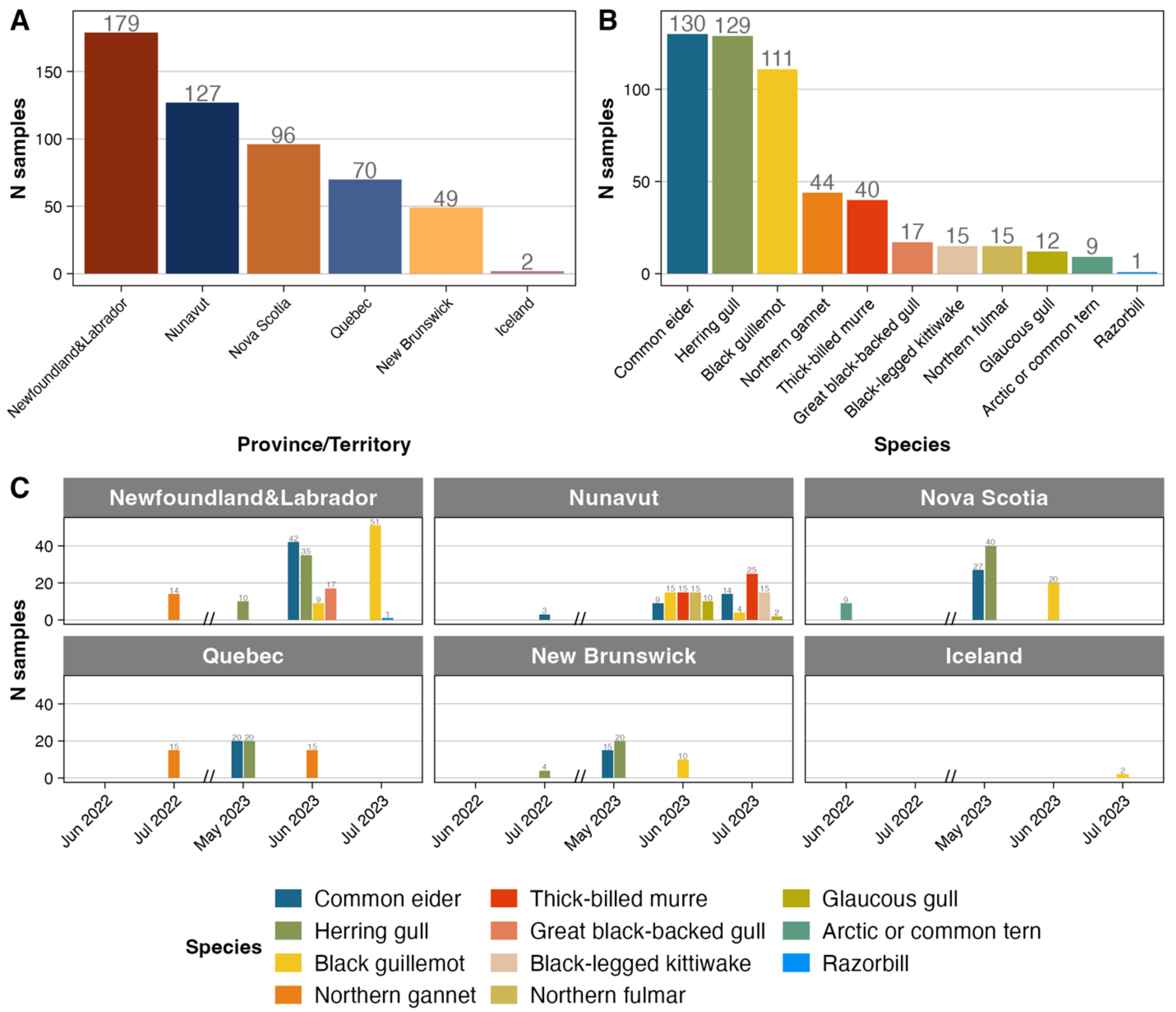

Fig. S2. Number (N) of samples collected to test for presence of AIV anti-NP and anti-H5 antibodies by **A)** province/territory/Iceland overall, **B)** species overall, and **C)** species within provinces/territories/Iceland by collection month.

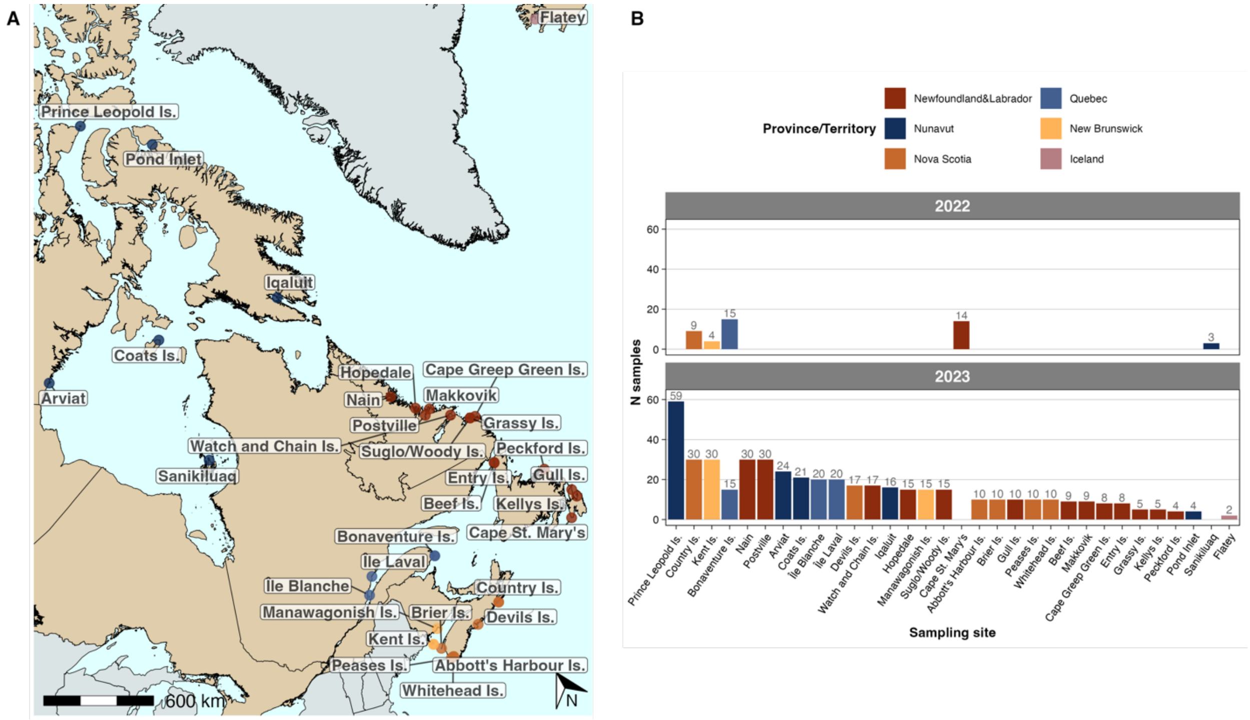

Fig. S3. Samples collected in 2022 and 2023 by site. **A)** Unique sites labeled and colored by province/territory/Iceland. **B)** Number of samples collected in 2022 and 2023 by site.

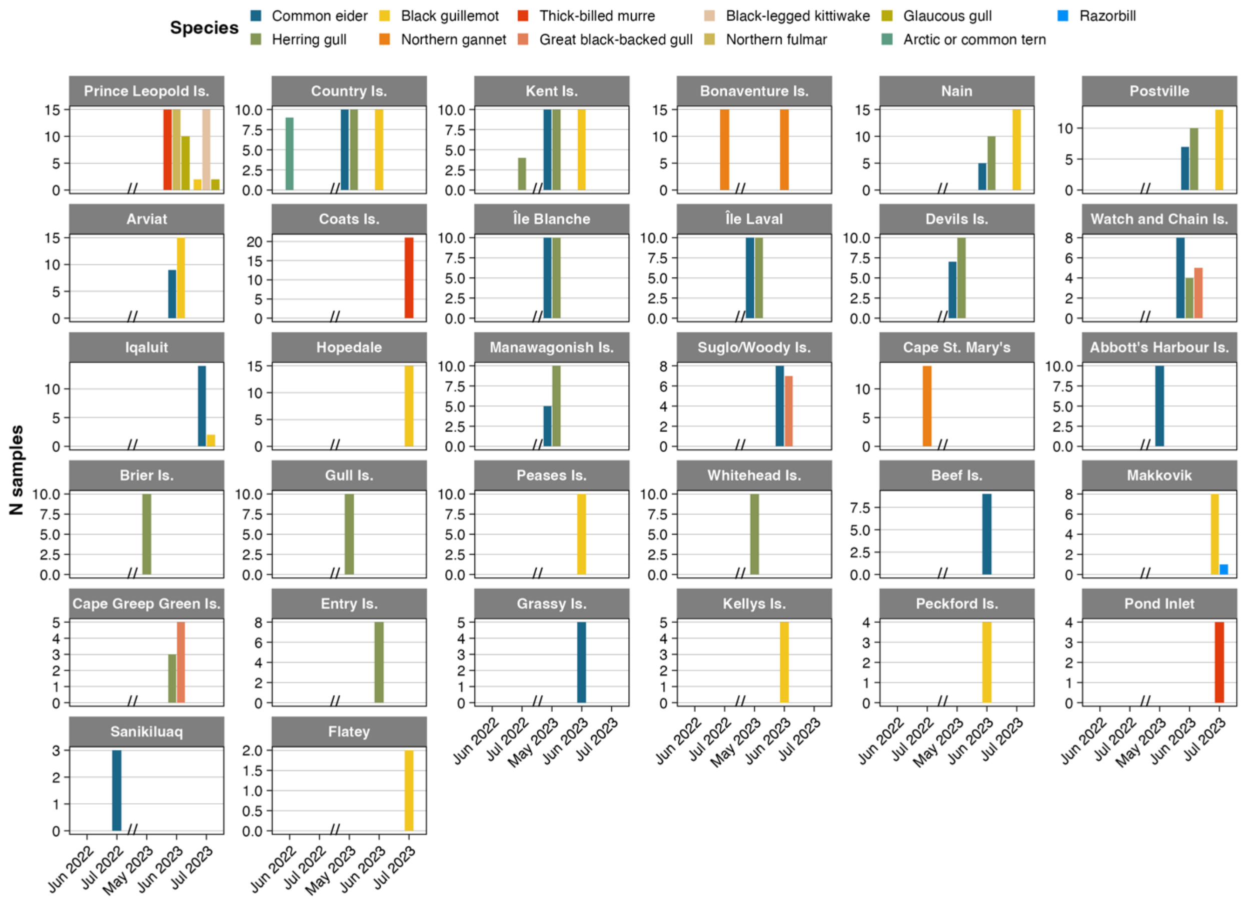

Fig. S4. Number of samples collected by month (x-axis), site (panel), and species (color).

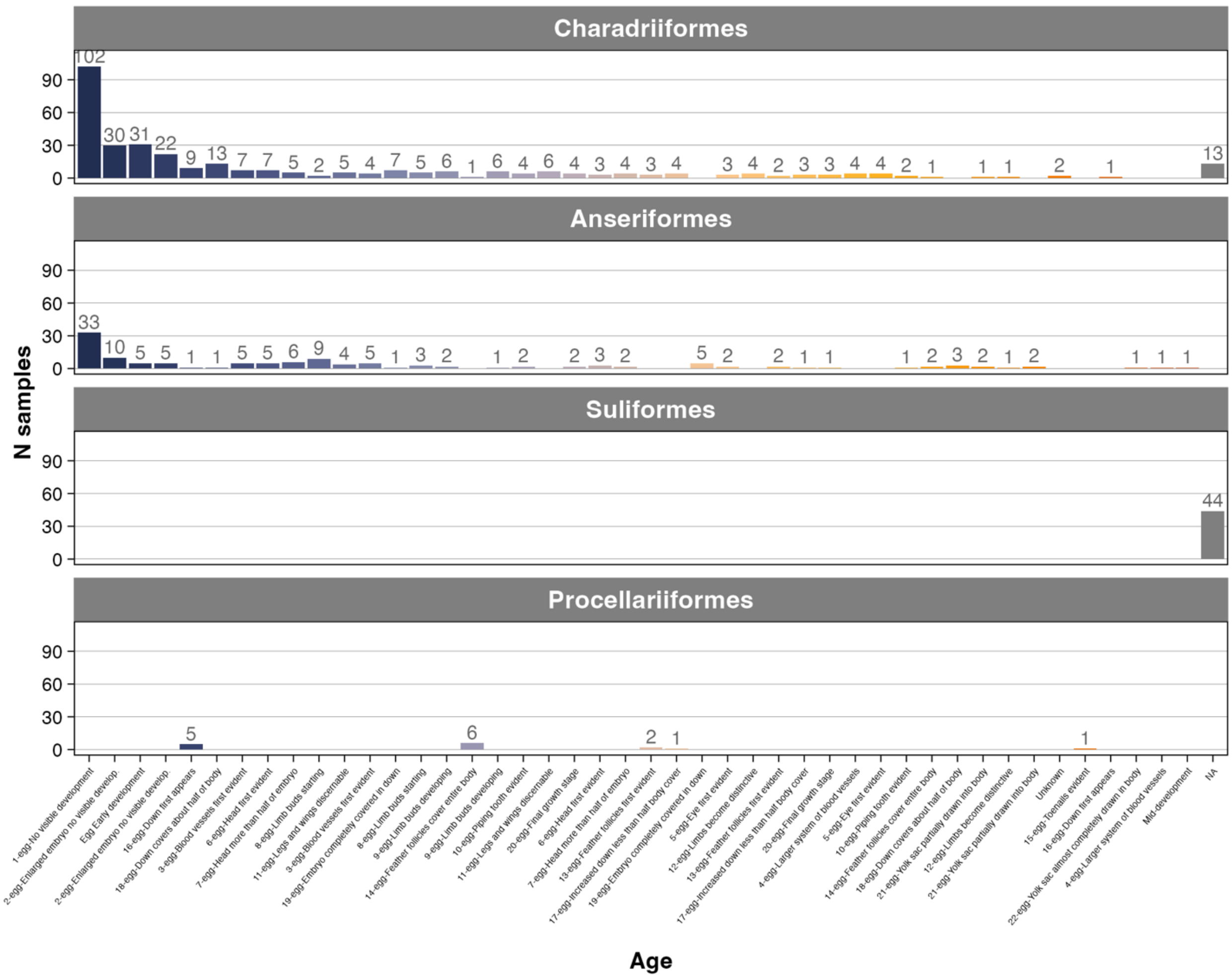

Fig. S5. Number of samples collected by egg developmental age and taxonomic order.

Assay evaluation to detect AIV antibodies in eggs

We evaluated the Biostone kits' precision and lower limit to detect AIV antibodies in egg samples processed at the NWRC (**Fig. S6)**. There was high intra-assay precision between duplicates for anti-NP antibodies (Pearson's correlation coefficient, r = 0.990) and anti-H5 antibodies (r = 0.987). Duplicate anti-H5 antibodies standard serial dilution series were used to explore the lower limit of detection (LOD). The relationship between $\log_{10}$(anti-H5 concentration) and $\log_{10}$(absorbance at OD_{450}) was linear above 12.5 ng/mL anti-H5 (**Fig. S7A**; adjusted $R^2 = 0.95$). Therefore, 12.5 ng/mL anti-H5 (corresponding to $OD_{450} = 1.316$) is an approximate lower LOD of this assay, near to the threshold PI ≥ 45 for anti-H5 detection recommended by the manufacturer (**Fig. S7B**).

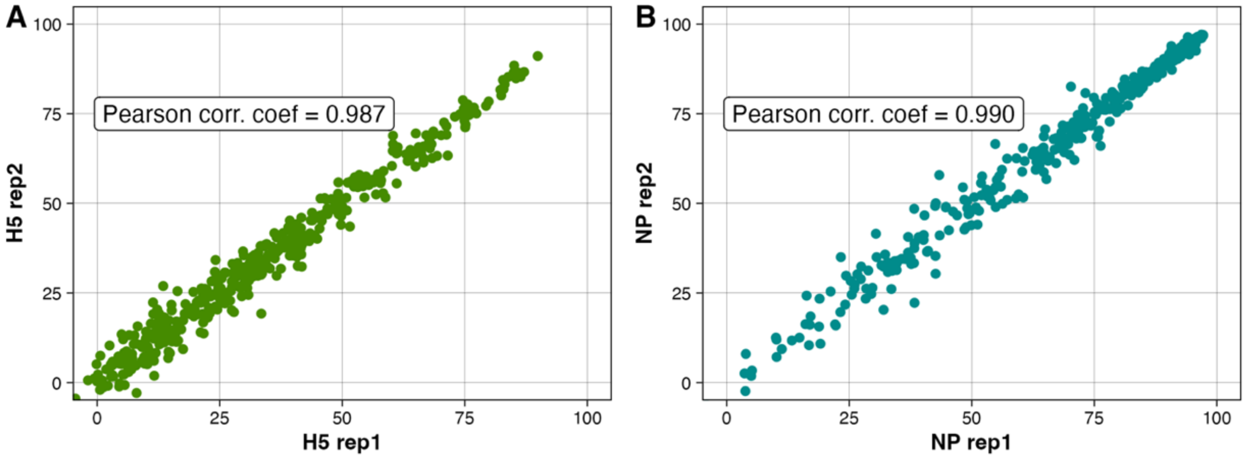

Fig. S6. Intra-assay precision of ELISA duplicates processed at NWRC with Biostone kits (*n*=491). Percentage inhibition (PI) of **A)** anti-H5 and **B)** anti-NP antibodies. Pearson's correlation coefficient reported.

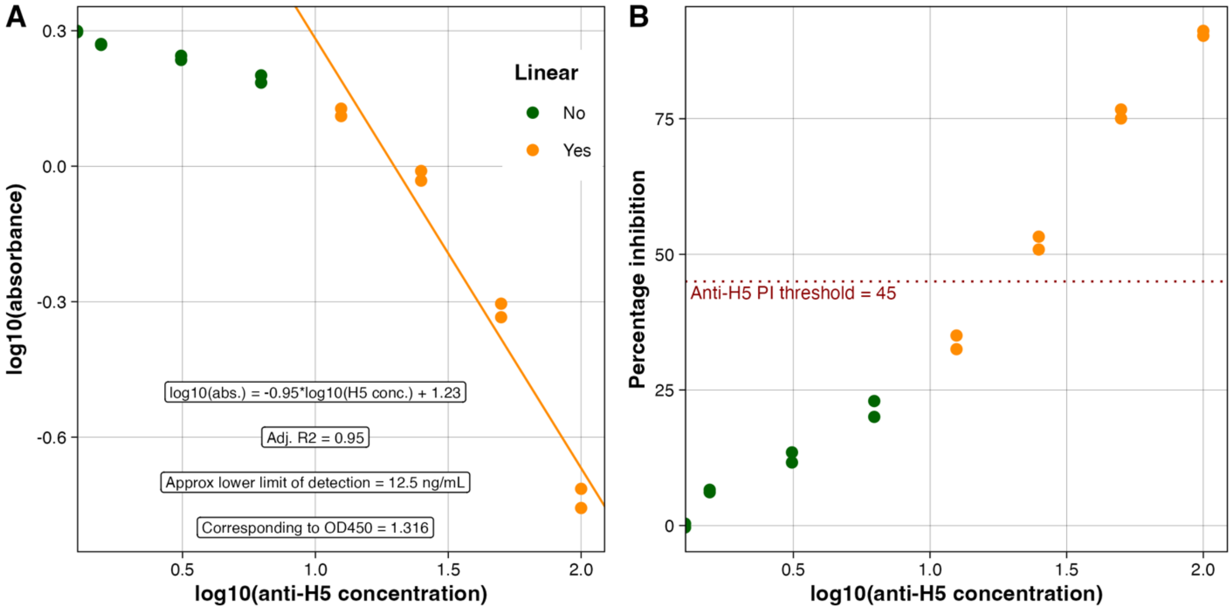

Fig. S7. Evaluation of Biostone lower limit of detection for anti-H5 antibody standard. **A)** The log-linear relationship between anti-H5 antibody concentration standard and absorbance (OD_{450}) in a competitive ELISA. Samples were run in duplicate. Data colored by linear range of the curve. **B)** Concentration of anti-H5 standard versus percentage inhibition (PI), colored by linear range. Annotation for recommend anti-H5 PI threshold.

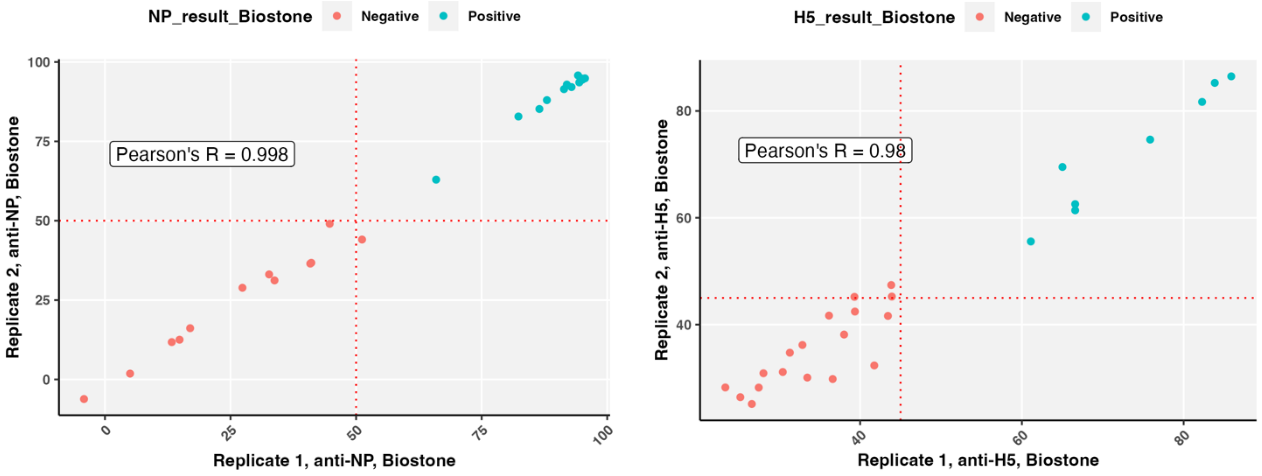

Fig. S8. Agreement between technical duplicates' percentage inhibition (PI) in Biostone anti-NP and anti-H5 antibody assays for 26 egg samples for which all ELISA protocols were compared. Pearson's correlation coefficient, r, reported. Red dashed lines indicate the replicate threshold for anti-NP, PI ≥ 50 and anti-H5, PI ≥ 45 for antibody detection.

Supplementary Tables

Table S1. Colonies/sites where eggs were collected for avian influenza serology testing and permit information. Permits and animal care certificates vary due to the collaborative nature of the program, the region the work takes place in, and the use of eggs collected for other programs being mobilized for this egg serology study. Latitude/longitude in decimal degree were rounded to three digits. Latitude/longitude for Iqaluit reflects the sampling location of common eider at Algerine Island and black guillemot sampled at Pitsiulaaq.

Colony/Site	Latitude, Longitude	Species	Permits	Animal Care
Abbott's Harbour Is., NS	43.661, -65.827	COEI	CWS Science ST2715	ECCC - 23ME01
Arviat, NU	61.108, -94.062	BLGU, COEI	CWS Science S C-NR-2023-NU-002 Nunavut Wildlife WL-2023-036	Community-based egg collections – no animal care required
Île Blanche, QC	47.929, -69.674	COEI, HERG	CWS Science SC-68 CWS National Wildlife Area RES-323	ECCC - 23ME01
Beef Island, NL	51.166, -56.813	COEI	CWS Science ST2715	ECCC - 23ME01
Bonaventure Island, QC	48.494, -64.161	NOGA	CWS Science SC-68	ECCC – 23JP01
Brier Island, NS	44.254, -66.368	HERG	CWS Science ST2715	ECCC - 23ME01
Cape Greep Green Island, NL	53.602, -56.153	GBBG, HERG	CWS Science ST2715	ECCC - 23ME01
Cape St Mary's, NL	46.823, -54.192	NOGA	NFLD Protected Areas CMSER_2022-23	ECCC – 23JP01
Coats Island, NU	62.950, -82.017	TBMU	CWS Science S C-NR-2023-NU-002 Nunavut Wildlife WL-2023-036	ECCC – 23JP01
Country Island, NS	45.102, -61.543	Tern (ARTE or COTE), BLGU, COEI, HERG	CWS Science ST2715	ECCC - 23ME01
Devils Island, NS	44.582, -63.459	HERG, COEI	CWS Science ST2715	ECCC - 23ME01
Entry Island, NL	51.142, -56.833	HERG	CWS Science ST2715	ECCC - 23ME01
Flatey, Iceland	65.374, -22.916	BLGU	Iceland export permit (no number provided)	NA
Grassy Island, NL	53.672, -56.575	COEI	CWS Science ST2715	ECCC - 23ME01
Gull Island, NL	47.955, -53.042	HERG	CWS Science ST2715	ECCC - 23ME01
Hopedale, NL	55.457, -60.212	BLGU	CWS Science SC4081	Community-based egg collections – no animal care required

			Nunatsiavut Government NGRAC-19834564	
Iqaluit, NU	63.458, -67.986	BLGU, COEI	CWS Science S C-NR-2023-NU-002<br>Nunavut Wildlife WL-2023-036	ECCC – 23JP01
Kellys Island, NL	47.547, -53.014	BLGU	CWS Science ST2715	ECCC - 23ME01
Kent Island, NB	44.583, -66.756	BLGU, COEI, HERG	CWS Science ST2715	ECCC - 23ME01
Île Laval, QC	48.752, -69.033	COEI HERG	CWS Science SC-68	ECCC - 23ME01
Makkovik, NL	55.088, -59.178	BLGU, RAZO	CWS Science SC4081<br>Nunatsiavut Government NGRAC-19834564	Community-based egg collections – no animal care required
Manawagonish Island, NB	45.208, -66.108	COEI, HERG	CWS Science ST2715	ECCC - 23ME01
Nain, NL	56.542, -61.697	BLGU, COEI, HERG	CWS Science SC4081<br>Nunatsiavut Government NGRAC-19834564	Community-based egg collections – no animal care required
Peases Island, NS	43.63, -66.03	BLGU	CWS Science ST2715	ECCC - 23ME01
Peckford Island, NL	49.541, -53.854	BLGU	CWS Science ST2715	ECCC - 23ME01
Pond Inlet, NU	72.683, -78.051	TBMU	CWS Science S C-NR-2023-NU-002<br>Nunavut Wildlife WL-2023-036	Community-based egg collections – no animal care required
Postville, NL	54.912, -59.762	BLGU, COEI, HERG	CWS Science SC4081<br>Nunatsiavut Government NGRAC-19834564	Community-based egg collections – no animal care required
Prince Leopold Island, NU	74.033, -90.061	TBMU, NOFU, BLGU, BLKI, GLGU	CWS Science S C-NR-2023-NU-002<br>CWS Protected Areas MM-NR-2023-NU-011<br>Government of Nunavut Wildlife WL-2023-036	ECCC – 23JP01
Sanikiluaq, NU	56.55, -79.185	COEI	CWS Science SC-NR-2021-NU-002<br>GN WL 2021-047	ECCC - 22GG02
Suglo/Woody Island, NL	53.666, -56.585	COEI, GBBG	CWS Science ST2715	ECCC - 23ME01
Watch and Chain Islands, NL	54.292, -57.869	COEI, GBBG, HERG	CWS Science ST2715<br>NGRAC-45303964	ECCC - 23ME01
Whitehead Island, NS	43.664, -65.867	HERG	CWS Science ST2715	ECCC - 23ME01

Table S2. Samples tested and positive detections of anti-NP and anti-H5 antibodies by region (province/territory/Iceland) and year (*2022 italicized*). The 95% confidence intervals (CI) were calculated using one-group two-sided proportions tests.

		Anti-NP			Anti-H5		
**Region**	**Year**	**N test**	**N pos.**	**% pos. (95% CI)**	**N test**	**N pos.**	**% pos. (95% CI)**
Newfoundland & Labrador (NL)	*2022*	14	0	0% (0-26.8%)	-	-	-
	2023	165	148	89.7% (83.8-93.7%)	148	32	21.6% (15.5-29.3%)
Nunavut (NU)	*2022*	3	3	100% (31-100%)	3	0	0% (0-69%)
	2023	124	93	75% (66.3-82.1%)	93	16	17.2% (10.4-26.7%)
Nova Scotia (NS)	*2022*	9	0	0% (0-37.1%)	-	-	-
	2023	87	57	65.5% (54.5-75.2%)	70	20	28.6% (18.7-40.8%)
Quebec (QC)	2022	15	0	0% (0-25.3%)	-	-	-
	2023	55	43	78.2% (64.6-87.8%)	43	25	58.1% (42.2-72.6%)
New Brunswick (NB)	*2022*	4	1	25% (1.3-78.1%)	1	0	0% (0-94.5%)
	2023	45	36	80% (64.9-89.9%)	45	11	24.4% (13.4-39.9%)
Iceland	2023	2	2	100% (19.8-100%)	2	0	0% (0-80.2%)

Table S3. Samples tested and positive detections of anti-NP and anti-H5 antibodies by site and year (*2022 italicized*).

			Anti-NP			Anti-H5		
**Sampling Site**	**Province/ Territory**	**Year**	**N test**	**N pos.**	**% pos. (95% CI)**	**N test**	**N pos.**	**% pos. (95% CI)**
Prince Leopold Is.	NU	2023	59	40	67.8% (54.2-79%)	40	9	22.5% (11.4-38.9%)
Country Is.	NS	*2022*	9	0	0% (0-37.1%)	-	-	-
	NS	2023	30	24	80% (60.9-91.6%)	25	9	36% (18.7-57.4%)
Kent Is.	NB	*2022*	4	1	25% (1.3-78.1%)	1	0	0% (0-94.5%)
	NB	2023	30	25	83.3% (64.5-93.7%)	30	9	30% (15.4-49.6%)
Bonaventure Is.	QC	*2022*	15	0	0% (0-25.3%)	-	-	-
	QC	2023	15	6	40% (17.5-67.1%)	6	3	50% (18.8-81.2%)
Nain	NL	2023	30	26	86.7% (68.4-95.6%)	26	3	11.5% (3-31.3%)
Postville	NL	2023	30	26	86.7% (68.4-95.6%)	26	2	7.7% (1.3-26.6%)
Arviat	NU	2023	24	18	75% (52.9-89.4%)	18	0	0% (0-21.9%)
Coats Is.	NU	2023	21	20	95.2% (74.1-99.8%)	20	3	15% (4-38.9%)
Île Blanche	QC	2023	20	17	85% (61.1-96%)	17	10	58.8% (33.5-80.6%)
Île Laval	QC	2023	20	20	100% (80-100%)	20	12	60% (36.4-80%)
Devils Is.	NS	2023	17	12	70.6% (44-88.6%)	12	8	66.7% (35.4-88.7%)
Watch and Chain Is.	NL	2023	17	15	88.2% (62.3-97.9%)	15	4	26.7% (8.9-55.2%)
Iqaluit	NU	2023	16	11	68.8% (41.5-87.9%)	11	4	36.4% (12.4-68.4%)
Hopedale	NU	2023	15	15	100% (74.7-100%)	15	0	0% (0-25.3%)
Manawagonish Is.	NB	2023	15	11	73.3% (44.8-91.1%)	15	2	13.3% (2.3-41.6%)
Suglo/Woody Is.	NL	2023	15	15	100% (74.7-100%)	15	6	40% (17.5-67.1%)
Cape St. Mary's	NL	2022	14	0	0% (0-26.8%)	-	-	-
Abbott's Harbour Is.	NS	2023	10	2	20% (3.5-55.8%)	10	3	30% (8.1-64.6%)
Brier Is.	NS	2023	10	9	90% (54.1-99.5%)	9	0	0% (0-37.1%)
Gull Is.	NL	2023	10	10	100% (65.5-100%)	10	1	10% (0.5-45.9%)
Peases Is.	NS	2023	10	4	40% (13.7-72.6%)	4	0	0% (0-60.4%)
Whitehead Is.	NS	2023	10	6	60% (27.4-86.3%)	10	0	0% (0-34.5%)
Beef Is.	NL	2023	9	9	100% (62.9-100%)	9	7	77.8% (40.2-96.1%)
Makkovik	NL	2023	9	8	88.9% (50.7-99.4%)	8	0	0% (0-40.2%)
Cape Greep Green Is.	NL	2023	8	8	100% (59.8-100%)	8	4	50% (21.5-78.5%)
Entry Is.	NL	2023	8	6	75% (35.6-95.5%)	6	1	16.7% (0.9-63.5%)
Grassy Is.	NL	2023	5	5	100% (46.3-100%)	5	4	80% (29.9-98.9%)
Kellys Is.	NL	2023	5	3	60% (17-92.7%)	3	0	0% (0-69%)
Peckford Is.	NL	2023	4	2	50% (15-85%)	2	0	0% (0-80.2%)
Pond Inlet	NU	2023	4	4	100% (39.6-100%)	4	0	0% (0-60.4%)
Sanikiluaq	NU	*2022*	3	3	100% (31-100%)	3	0	0% (0-69%)
Flatey	Iceland	2023	2	2	100% (19.8-100%)	2	0	0% (0-80.2%)

Table S4. Samples tested and positive detections of anti-NP and anti-H5 antibodies by species and year (*2022 italicized*).

Species		Anti-NP			Anti-H5		
	**Year**	**N test**	**N pos.**	**% pos. (95% CI)**	**N test**	**N pos.**	**% pos. (95% CI)**
Herring gull	*2022*	4	1	25% (1.3-78.1%)	1	0	0% (0-94.5%)
	2023	125	92	73.6% (64.8-80.9%)	104	8	7.7% (3.6-15%)
Common eider	*2022*	3	3	100% (31-100%)	3	0	0% (0-69%)
	2023	127	109	85.8% (78.3-91.2%)	119	74	62.2% (52.8-70.8%)
Black guillemot	2023	111	93	83.8% (75.3-89.9%)	93	1	1.1% (0.1-6.7%)
Northern gannet	*2022*	29	0	0% (0-14.6%)	-	-	-
	2023	15	6	40% (17.5-67.1%)	6	3	50% (18.8-81.2%)
Thick-billed murre	2023	40	39	97.5% (85.3-99.9%)	39	8	20.5% (9.9-36.9%)
Great black-backed gull	2023	17	16	94.1% (69.2-99.7%)	16	6	37.5% (16.3-64.1%)
Black-legged kittiwake	2023	15	6	40% (17.5-67.1%)	6	0	0% (0-48.3%)
Northern fulmar	2023	15	8	53.3% (27.4-77.7%)	8	0	0% (0-40.2%)
Glaucous gull	2023	12	10	83.3% (50.9-97.1%)	10	4	40% (13.7-72.6%)
Arctic or common tern	2022	9	0	0% (0-37.1%)	-	-	-
Razorbill	2023	1	0	0% (0-94.5%)	-	-	-

Table S5. Samples tested and positive detections of anti-NP and anti-H5 antibodies by site, species, and year (*2022 italicized*). Anti-NP and anti-H5 ELISA protocols reported.

				Anti-NP				Anti-H5			
**Region**	**Site**	**Species**	**Year**	**N test**	**n pos.**	**% pos. (95% CI)**	**Assay**	**N test**	**N pos.**	**% pos. (95% CI)**	**Assay**
NL	Nain	Herring gull	2023	10	8	80% (44.2-96.5%)	Biostone	10	1	10% (0.5-45.9%)	Biostone
		Common eider	2023	5	5	100% (46.3-100%)	IDEXX	5	2	40% (7.3-83%)	NCFAD
		Black guillemot	2023	15	13	86.7% (58.4-97.7%)	Biostone	15	0	0% (0-25.3%)	Biostone
	Postville	Herring gull	2023	10	6	60% (27.4-86.3%)	Biostone	10	0	0% (0-34.5%)	Biostone
		Common eider	2023	7	7	100% (56.1-100%)	Biostone	7	2	28.6% (5.1-69.7%)	Biostone
		Black guillemot	2023	13	13	100% (71.7-100%)	Biostone	13	0	0% (0-28.3%)	Biostone
	Watch and Chain Is.	Herring gull	2023	4	4	100% (39.6-100%)	Biostone	4	1	25% (1.3-78.1%)	Biostone
		Common eider	2023	8	7	87.5% (46.7-99.3%)	Biostone	8	3	37.5% (10.2-74.1%)	Biostone
		Great black-backed gull	2023	5	4	80% (29.9-98.9%)	Biostone	5	0	0% (0-53.7%)	Biostone
	Hopedale	Black guillemot	2023	15	15	100% (74.7-100%)	Biostone	15	0	0% (0-25.3%)	Biostone
	Suglo/ Woody Is.	Common eider	2023	8	8	100% (59.8-100%)	Biostone	8	4	50% (21.5-78.5%)	Biostone
		Great black-backed gull	2023	7	7	100% (56.1-100%)	Biostone	7	2	28.6% (5.1-69.7%)	Biostone
	Cape St. Mary's	Northern gannet	*2022*	14	0	0% (0-26.8%)	IDEXX	14	0	0% (0-26.8%)	NCFAD
	Gull Is.	Herring gull	2023	10	10	100% (65.5-100%)	Biostone	10	1	10% (0.5-45.9%)	Biostone
	Beef Is.	Common eider	2023	9	9	100% (62.9-100%)	Biostone	9	7	77.8% (40.2-96.1%)	Biostone
	Makkovik	Black guillemot	2023	8	8	100% (59.8-100%)	Biostone	8	0	0% (0-40.2%)	Biostone
		Razorbill	2023	1	0	0% (0-94.5%)	Biostone	1	0	0% (0-94.5%)	Biostone
	Cape Greep Green Is.	Herring gull	2023	3	3	100% (31-100%)	Biostone	3	0	0% (0-69%)	Biostone
		Great black-backed gull	2023	5	5	100% (46.3-100%)	Biostone	5	4	80% (29.9-98.9%)	Biostone
	Entry Is.	Herring gull	2023	8	6	75% (35.6-95.5%)	Biostone	8	1	12.5% (0.7-53.3%)	Biostone
	Grassy Is.	Common eider	2023	5	5	100% (46.3-100%)	Biostone	5	4	80% (29.9-98.9%)	Biostone
	Kellys Is.	Black guillemot	2023	5	3	60% (17-92.7%)	Biostone	5	0	0% (0-53.7%)	Biostone
	Peckford Is.	Black guillemot	2023	4	2	50% (15-85%)	Biostone	4	0	0% (0-60.4%)	Biostone
NU	Prince Leopold Is.	Black guillemot	2023	2	1	50% (9.5-90.5%)	Biostone	2	0	0% (0-80.2%)	Biostone
		Thick-billed murre	2023	15	15	100% (74.7-100%)	Biostone	15	5	33.3% (13-61.3%)	Biostone
		Black-legged kittiwake	2023	15	6	40% (17.5-67.1%)	Biostone	15	0	0% (0-25.3%)	Biostone
		Northern fulmar	2023	15	8	53.3% (27.4-77.7%)	Biostone	15	0	0% (0-25.3%)	Biostone
		Glaucous gull	2023	12	10	83.3% (50.9-97.1%)	Biostone	12	4	33.3% (11.3-64.6%)	Biostone
	Arviat	Common eider	2023	9	7	77.8% (40.2-96.1%)	Biostone	9	0	0% (0-37.1%)	Biostone
		Black guillemot	2023	15	11	73.3% (44.8-91.1%)	Biostone	15	0	0% (0-25.3%)	Biostone
	Coats Is.	Thick-billed murre	2023	21	20	95.2% (74.1-99.8%)	Biostone	21	3	14.3% (3.8-37.4%)	Biostone
	Iqaluit	Common eider	2023	14	9	64.3% (35.6-86%)	Biostone	14	4	28.6% (9.6-58%)	Biostone
		Black guillemot	2023	2	2	100% (19.8-100%)	Biostone	2	0	0% (0-80.2%)	Biostone
	Pond Inlet	Thick-billed murre	2023	4	4	100% (39.6-100%)	Biostone	4	0	0% (0-60.4%)	Biostone
	Sanikiluaq	Common eider	*2022*	3	3	100% (31-100%)	Biostone	3	0	0% (0-69%)	Biostone
NS	Country Is.	Herring gull	2023	10	6	60% (27.4-86.3%)	Biostone	10	0	0% (0-34.5%)	Biostone
		Common eider	2023	10	9	90% (54.1-99.5%)	Biostone	10	8	80% (44.2-96.5%)	Biostone
		Black guillemot	2023	10	9	90% (54.1-99.5%)	Biostone	10	1	10% (0.5-45.9%)	Biostone
		Arctic, common Tern	*2022*	9	0	0% (0-37.1%)	IDEXX	9	0	0% (0-37.1%)	NCFAD
	Devils Is.	Herring gull	2023	10	5	50% (23.7-76.3%)	Biostone	10	1	10% (0.5-45.9%)	Biostone
		Common eider	2023	7	7	100% (56.1-100%)	Biostone	7	7	100% (56.1-100%)	Biostone
	Abbott's Harbour Is.	Common eider	2023	10	2	20% (3.5-55.8%)	Biostone	10	3	30% (8.1-64.6%)	Biostone
	Brier Is.	Herring gull	2023	10	9	90% (54.1-99.5%)	Biostone	10	0	0% (0-34.5%)	Biostone
	Peases Is.	Black guillemot	2023	10	4	40% (13.7-72.6%)	Biostone	10	0	0% (0-34.5%)	Biostone
	Whitehead Is.	Herring gull	2023	10	6	60% (27.4-86.3%)	Biostone	10	0	0% (0-34.5%)	Biostone
QC	Bonaventure Is.	Northern gannet	*2022*	15	0	0% (0-25.3%)	IDEXX	15	0	0% (0-25.3%)	NCFAD
			2023	15	6*	40% (17.5-67.1%)	IDEXX,	15	3	20% (5.3-48.6%)	NCFAD
	Île Blanche	Herring gull	2023	10	7	70% (35.4-91.9%)	Biostone	10	0	0% (0-34.5%)	Biostone
		Common eider	2023	10	10	100% (65.5-100%)	Biostone	10	10	100% (65.5-100%)	Biostone
	Île Laval	Herring gull	2023	10	10	100% (65.5-100%)	Biostone	10	2	20% (3.5-55.8%)	Biostone
		Common eider	2023	10	10	100% (65.5-100%)	Biostone	10	10	100% (65.5-100%)	Biostone
NB	Kent Is.	Herring gull	*2022*	4	1	25% (1.3-78.1%)	IDEXX	1	0	0% (0-94.5%)	NCFAD
			2023	10	5	50% (23.7-76.3%)	Biostone	10	1	10% (0.5-45.9%)	Biostone
		Common eider	2023	10	10	100% (65.5-100%)	Biostone	10	8	80% (44.2-96.5%)	Biostone
		Black guillemot	2023	10	10	100% (65.5-100%)	Biostone	10	0	0% (0-34.5%)	Biostone
	Manawagonish Is.	Herring gull	2023	10	7	70% (35.4-91.9%)	Biostone	10	0	0% (0-34.5%)	Biostone
		Common eider	2023	5	4	80% (29.9-98.9%)	Biostone	5	2	40% (7.3-83%)	Biostone
Iceland	Flatey	Black guillemot	2023	2	2	100% (19.8-100%)	Biostone	2	0	0% (0-80.2%)	Biostone
